# Analyzing Big Data with the Hybrid Interval Regression Methods

**DOI:** 10.1155/2014/243921

**Published:** 2014-07-20

**Authors:** Chia-Hui Huang, Keng-Chieh Yang, Han-Ying Kao

**Affiliations:** ^1^Department of Business Administration, National Taipei University of Business, No. 321, Section 1, Jinan Road, Zhongzheng District, Taipei City 100, Taiwan; ^2^Department of Information Management, Hwa Hsia Institute of Technology, No. 111, Gongzhuan Road, Zhonghe District, New Taipei City 235, Taiwan; ^3^Department of Computer Science and Information Engineering, National Dong Hwa University, No. 123, Hua-Shi Road, Hualien 97063, Taiwan

## Abstract

Big data is a new trend at present, forcing the significant impacts on information technologies. In big data applications, one of the most concerned issues is dealing with large-scale data sets that often require computation resources provided by public cloud services. How to analyze big data efficiently becomes a big challenge. In this paper, we collaborate interval regression with the smooth support vector machine (SSVM) to analyze big data. Recently, the smooth support vector machine (SSVM) was proposed as an alternative of the standard SVM that has been proved more efficient than the traditional SVM in processing large-scale data. In addition the soft margin method is proposed to modify the excursion of separation margin and to be effective in the gray zone that the distribution of data becomes hard to be described and the separation margin between classes.

## 1. Introduction

Big data has become one of new research frontiers. Generally speaking, big data is a collection of large-scale and complex data sets that it becomes more difficult to process using current database management systems and traditional data processing applications. In 2012, Gartner Inc. gave a definition of big data as “Big data is high volume, high velocity, and/or high variety information assets that require new forms of processing to enable enhanced decision making, insight discovery and process optimization” [1]. The trend of big data sets is due to the additional information derivable from analysis of a single large set of related data, as compared to separate smaller sets with the same total amount of data.

One of the major applications of the future parallel, distributed, and cloud systems is in big data analytic [2–5]. Most concerned issues are dealing with large-scale sets which often require computation resources provided by public cloud services. How to analyze big data efficiently becomes a big challenge.

The support vector machine (SVM) has shown to be an efficient approach for a variety of data mining, classification, analysis, pattern recognition, and distribution estimation [6–14]. Recently, using SVM to solve the interval regression model [15] has become an alternative approach. Hong and Hwang [16] evaluated interval regression models with quadratic loss SVM. Bisserier et al. [17] proposed a revisited fuzzy regression method where a linear model is identified from Crisp-Inputs Fuzzy-Outputs (CISO) data. D'Urso et al. [18] presented fuzzy clusterwise regression analysis with LR fuzzy response variable and numeric explanatory variables. The suggested model is to allow for linear and nonlinear relationship between the output and input variables. Jeng et al. [19] developed a support vector interval regression networks (SVIRNs) based on both SVM and neural networks. Huang and Kao [20] proposed a soft-margin SVM for interval regression analysis. Huang [21] solved interval regression model with reduced support vector machine.

However, there are several main problems while using SVM model.

1. Big data: when dealing with big data sets, the solution by using SVM with a nonlinear kernel may be difficult to be found.
2. Noises and interaction: the distribution of data becomes hard to be described and the separation margin between classes becomes a “gray” zone.
3. Unbalance: the number of samples from one class is much larger than the number of samples from other classes. It causes the excursion of separation margin.

Under this circumstance, developing an efficient method to analyze big data becomes important. The smooth support vector machine (SSVM) has been proved more efficient than the traditional SVM in processing large-scale data [22–24]. The main idea of SSVM is solved by a fast Newton-Armijo algorithm [25] and has been extended to nonlinear separation surfaces by using a nonlinear kernel technology [24].

In this study, we collaborate interval regression [15] with SSVM to analyze big data. The main idea of SSVM is solved by a fast Newton-Armijo algorithm and has been extended to nonlinear separation surfaces by using a nonlinear kernel technology. Additionally, to modify the excursion of separation margin and to be effective in the gray zone, the soft margin method is proposed. The experiment results show that the proposed methods are more efficient than existing methods.

This study is organized as follows.  Section 2 reviews the current methods for interval regression analysis.  Section 3 proposes the soft margin method and the formulation of interval regression with SSVM to analyze big data.  Section 4 gives a numerical example by the proposed methods dealing with big data which is extracted from Taiwan Stock Exchange Capitalization Weighted Stock Index (TAIEX) [26]. Finally, Section 5 gives the concluding remarks.

## 2. Literature Review

Since Tanaka et al. [27] introduced the fuzzy regression model with symmetric fuzzy parameters, the properties of fuzzy regression have been studied extensively by many researchers. Fuzzy regression model can be simplified to interval regression analysis which is considered as the simplest version of possibilistic regression analysis with interval coefficients. An interval linear regression model is described as

(1) $$\begin{array}{c} Y\left(x_{j}\right)=A_{0}+A_{1}x_{1j}+\cdots +A_{n}x_{nj}, \end{array}$$

where *Y*(**x**_*j*_), *j* = 1,2,…, *q*, is the estimated interval corresponding to the real input vector **x**_*j*_ = (*x*_1*j*_, *x*_2*j*_,…, *x*_*nj*_)^*t*^. An interval coefficient *A*_*i*_ is defined as (*a*_*i*_, *c*_*i*_), where *a*_*i*_ is the center and *c*_*i*_ is the radius. Hence, *A*_*i*_ can also be represented as

(2) $$\begin{array}{c} A_{i}=\left[a_{i}-c_{i},a_{i}+c_{i}\right]=\left\{a_{i}-c_{i}\leq a\leq a_{i}+c_{i}\right\}. \end{array}$$

The interval linear regression model (1) can also be expressed as

(3) Y(xj)=A0+A1x1j+⋯+Anxnj=(a0,c0)+(a1,c1)x1j+⋯+(an,cn)xnj=(a0+∑i=1naixij,c0+∑i=1nci|xij|).

For a data set with crisp inputs and interval outputs, two interval regression models, the possibility and necessity models, are considered. By assumption, the center coefficients of the possibility regression model and the necessity regression model are the same [15]. For this data set, the possibility and necessity estimation models are defined as

(4) Y∗(xj)=A0∗+A1∗x1j+⋯+An∗xnjY∗(xj)=A0∗+A1∗x1j+⋯+An∗xnj,

where the interval coefficients *A*_i_* and *A*_*i*∗_ are defined as *A*_i_* = (*a*_i_*, *c*_i_*) and *A*_*i*∗_ = (*a*_*i*∗_, *c*_*i*∗_), respectively. The interval Y*(**x**_*j*_) estimated by the possibility model must include the observed interval *Y*_*j*_ and the interval *Y*_∗_(**x**_*j*_) estimated by the necessity model must be included in the observed interval *Y*_*j*_.

In this section, we review the current methods which are ordinarily used for interval regression analysis.

### 2.1. Tanaka and Lee's Approach

Tanaka and Lee [15] proposed an interval regression analysis with a quadratic programming (QP) approach which gives more diverse spread coefficients than a linear programming (LP) one.

The interval regression analysis by QP approach unifying the possibility and necessity models subject to the inclusion relations, *Y*_∗_(**x**_*j*_)⊆*Y*_*j*_⊆Y*(**x**_*j*_), can be represented as

(5) min⁡ ∑j=1q(d0+∑i=1ndi|xij|)2+φ∑i=0n(ai2+ci2)s.t. Y∗(xj)⊆Yj⊆Y∗(xj), j=1,2,…,qci,di≥0, i=0,1,…,n,

where *φ* is an extremely small positive number and makes the influence of the term *φ*∑_*i*=0_^*n*^(*a*_*i*_^2^ + *c*_*i*_^2^) on the objective function negligible. The constraints of the inclusion relations are equivalent to

(6) Y∗(xj)⊆Yj⟺{yj−ej≤(a0+∑i=1naixij)−(c0+∑i=1nci|xij|)(a0+∑i=1naixij)+(c0+∑i=1nci|xij|)≤yj+ej,

(7) Yj⊆Y∗(xj)⟺{(a0+∑i=1naixij)−(c0+∑i=1nci|xij|)  −(d0+∑i=1ndi|xij|)≤yj−ejyj+ej≤(a0+∑i=1naixij)+(c0+∑i=1nci|xij|)+(d0+∑i=1ndi|xij|),

where **x**_*j*_ is the *j*th input vector and *Y*_*j*_ is the corresponding interval output that consists of a center *y*_*j*_ and a radius *e*_*j*_ denoted by *Y*_*j*_ = (*y*_*j*_, *e*_*j*_).

### 2.2. Hong and Hwang's Approach

Hong and Hwang [16] evaluated interval regression model combining the possibility and necessity estimation formulation with the principle of quadratic loss support vector machine (QLSVM). This version of SVM utilizes the quadratic loss function. The QLSVM performs interval nonlinear regression analysis by constructing an interval linear regression function in high-dimensional feature space.

With the principle of QLSVM, the interval nonlinear regression model is given as follows:

(8) max⁡   −12(∑i,j=1n(λ2i−λ2i∗)(λ2j−λ2j∗)K(xi,xj)+∑i,j=1n(λ3i−λ3i∗)(λ3j−λ3j∗)K(xi,xj)+∑i,j=1n(λ4i−λ4i∗)(λ4j−λ4j∗)K(xi,xj)+2∑i,j=1n(λ2i−λ2i∗)(λ3j−λ3j∗)K(xi,xj)−2∑i,j=1n(λ2i−λ2i∗)(λ4j−λ4j∗)K(xi,xj)−2∑i,j=1n(λ3i−λ3i∗)(λ4j−λ4j∗)K(xi,xj)+∑i,j=1n(λ3i+λ3i∗)(λ3j+λ3j∗)K(|xi|,|xj|)+∑i,j=1n(λ4i+λ4i∗)(λ4j+λ4j∗)K(|xi|,|xj|)−2∑i,j=1n(λ3i+λ3i∗)(λ4j+λ4j∗)K(|xi|,|xj|)+∑i,j=1nλ1iλ1jK(|xi|,|xj|)−2∑i,j=1nλ1i(λ3j+λ3j∗)K(|xi|,|xj|))−12C∑i=1nλ1i2−12C∑i=1n(λ2i2+λ2i∗2)+∑i=1n(λ2i−λ2i∗)yi+∑i=1n(λ3i−λ3i∗)yi−∑i=1n(λ4i−λ4i∗)yi+∑i=1n(λ3i+λ3i∗)ϵi−∑i=1n(λ4i+λ4i∗)ϵis.t.   λ1i,λ2i,λ2i∗,λ3i,λ3i∗,λ4i,λ4i∗≥0,

where *λ*_1*i*_, *λ*_2*i*_, *λ*_2i_*, *λ*_3*i*_, *λ*_3i_*, *λ*_4*i*_, and *λ*_4i_* are Lagrange multipliers. *K*(∗) is a nonlinear kernel. The followings are well-known nonlinear kernels, where *σ*, *γ*, *r*, *h*, and *θ* are kernel parameters:

1. Gaussian (radial basis) kernel: *e* ^−||*x*_*i*_−*x*_*j*_||^2^/2*σ*^2^^, *σ* > 0 [10],
2. hyperbolic tangent kernel: tanh⁡(*γx*_*i*_ *x*_*j*_^*t*^ + *θ*), *γ* > 0 [12],
3. polynomial kernel: (*γx*_*i*_ *x*_*j*_^*t*^ + *r*)^*h*^, *h* ∈ *N*, *γ* > 0, and *r* ≥ 0 [14].

The advantage of Hong and Hwang's approach is a model-free method in the sense that there is no need to assume the underlying model function for interval nonlinear regression model with crisp inputs and interval output.

### 2.3. Huang's Approach

There are two problems while using the traditional SVM model. (1) Large scale: when dealing with large-scale data sets, the solution may be difficult to be found when using SVM with nonlinear kernels; (2) Unbalance: the number of samples from one class is much larger than the number of samples from the other classes. It causes the excursion of separation margin.

To resolve these problems, Huang [21] proposed a reduced support vector machine (RSVM) approach in evaluating interval regression models. RSVM has been proven more efficient than the traditional SVM in processing large-scale data.

With the principle of RSVM, the interval nonlinear regression model is listed as follows:

(9) max⁡ −12∑i,j=1nλ1iλ1jQ·,KtQ·,K−12∑i,j=1n(λ2i−λ2i∗)(λ2j−λ2j∗)K(xi,xj)−12∑i,j=1n(λ3i−λ3i∗)(λ3j−λ3j∗)K(xi,xj)−∑i,j=1nλ1iQ·,K(λ2j−λ2j∗)xj+∑i,j=1nλ1iQ·,K(λ3j−λ3j∗)xj+∑i,j=1n(λ2i−λ2i∗)(λ3j−λ3j∗)K(xi,xj)−12∑i,j=1n(λ2i+λ2i∗)(λ2j+λ2j∗)K(|xi|,|xj|)+∑i,j=1n(λ2i+λ2i∗)(λ3j+λ3j∗)K(|xi|,|xj|)−∑i,j=1n(λ3i+λ3i∗)(λ3j+λ3j∗)K(|xi|,|xj|)−14C∑i=1nλ1i2+∑i=1n(λ2i−λ2i∗)yi−∑i=1n(λ3i−λ3i∗)yi−∑i=1n(λ2i+λ2i∗)ϵi+∑i=1n(λ3i+λ3i∗)ϵis.t. λ1i,λ2i,λ2i∗,λ3i,λ3i∗≥0,

where *λ*_1*i*_, *λ*_2*i*_, *λ*_2i_*, *λ*_3*i*_, and *λ*_3i_* are Lagrange multipliers. *Q* is a positive semidefinite matrix in RSVM. *K*(∗) is a nonlinear kernel.

The advantage of Huang's approach is to reduce the number of support vectors by randomly selecting a subset of samples. While processing with large-scale data sets, the solution can be found easily by the proposed method with nonlinear kernels.

## 3. Proposed Methods

In this section we first propose the soft margin method to modify the excursion of separation margin and to be effective in the gray zone. Then the formulation of interval regression with SSVM to analyze big data is introduced.

### 3.1. Soft Margin

In a conventional SVM, the sign function is used as the decision-making function. The separation threshold of the sign function is 0, which results in an excursion of separation margin for unbalanced data sets. The aim of the hard-margin separation margin is to find a hyperplane with the largest distance to the nearest training data. However, the limitations of the hard-margin formulation are as follows:

1. there is no separating hyperplane for certain training data;
2. complete separation with zero training error will lead to suboptimal prediction error;
3. it is difficult to deal with the gray zone between classes.

Thus, the soft margin method is proposed to modify the excursion of separation margin and to be effective in the gray zone. The soft margin is defined as

(10) $$\begin{array}{c} f^{-}\left(\delta \right)=\frac{\arctan\left(-\delta \cdot s+\vartheta \cdot s\right)}{\pi }+0.5,\\ f^{+}\left(\delta \right)=\frac{\arctan\left(\delta \cdot s-\vartheta \cdot s\right)}{\pi }+0.5, \end{array}$$

where *δ* is the decision value. *ϑ* and *s* are offset parameter and scale parameter which need to be estimated using statistical method.

**Figure 1:**
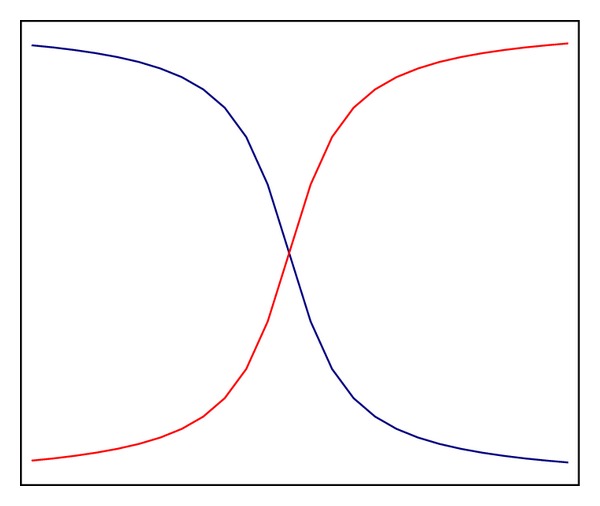
Soft margin.

With the soft margin as shown in Figure 1, the predication of the class labels can be determined as follows:

(11) y(x)={−1,if  (νr<f−(δ),δ<ϑ)  or  (νr>f+(δ),δ>ϑ)+1,if  (νr>f−(δ),δ<ϑ)  or  (νr<f+(δ),δ>ϑ),

where *ν*_*r*_ is a random number between 0 and 1.

### 3.2. Interval Regression with SSVM

The main idea of smooth support vector machine (SSVM) is solved by a fast Newton-Armijo algorithm [25] and has been extended to nonlinear separation surfaces by using a nonlinear kernel technology [24].

Suppose that *m* training data {*x*_*i*_, *y*_*i*_}, *i* = 1,2,…, *m*, are given, where *x*_*i*_ ∈ *R* ^*n*^ are the input patterns and *y*_*i*_ ∈ {−1,1} are the related target values of two-class pattern classification case. Then the standard support vector machine with a linear kernel [14] is

(12) min⁡w,b,ξ 12||w||2+C∑i=1mξi2s.t. yi(wtxi+b)≥1−ξiξi≥0, i=1,2,…,m,

where *b* is the location of hyperplane relative to the origin. The regularization constant *C* is a positive parameter to control the tradeoff between the training error and the part of maximizing the margin that is achieved by minimizing ||*w*||^2^. *ξ*_*i*_ is the slack variable with weight *C*/2. ||*w*|| is the Euclidean norm of *w* which is the normal to the following hyperplanes:

(13) $$\begin{array}{c} w^{t}x_{i}+b=+1,\;\text{for}\;\;y_{i}=+1, \end{array}$$

(14) $$\begin{array}{c} w^{t}x_{i}+b=-1,\;\text{for}\;\;y_{i}=-1. \end{array}$$

The first hyperplane (13) bounds the class {+1} and the second hyperplane (14) bounds the class {−1}. The linear separating hyperplane is

(15) $$\begin{array}{c} w^{t}x_{i}+b=0. \end{array}$$

In Lee and Mangasarian's approach [24], *b* ^2^/2 is added to the objective function of (12). This is equivalent to adding a constant feature to the training data and finding a separating hyperplane through the origin. Consider

(16) min⁡w,b,ξ 12(||w||2+b2)+C2∑i=1mξi2s.t. yi(wtxi+b)≥1−ξiξi≥0, i=1,2,…,m,

where *ξ*_*i*_ = {1 − *y*_*i*_(*w* ^*t*^ *x*_*i*_ + *b*)}_+_ for all *i* and the “+” function is defined as *x*_+_ = max⁡{0, *x*}. Then (12) can be reformulated as the following minimization problem by replacing *ξ*_*i*_ with {1 − *y*_*i*_(*w* ^*t*^ *x*_*i*_ + *b*)}_+_:

(17) $$\begin{array}{c} \underset{w,b}{\min}\;\frac{1}{2}\left({||w||}^{2}+b^{2}\right)+\frac{C}{2}\overset{m}{\underset{i=1}{\sum }}{\left\{1-y_{i}\left(w^{t}x_{i}+b\right)\right\}}_{+}^{2}. \end{array}$$

The objective function in (17) is not twice differentiable and can be solved by using a fast Newton-Armijo method [25]. Thus the “+” function in SSVM is approximated by a smooth function, *p*(*x*, *α*), as follows:

(18) $$\begin{array}{c} p\left(x,\alpha \right)=x+\frac{1}{\alpha }\log\left(1+e^{-\alpha x}\right),\;\alpha >0, \end{array}$$

where *α* > 0 is the smooth parameter. 1/(1 + *e* ^−*αx*^) is the integral of the sigmoid function of neural networks [28]. The *p*(*x*, *α*) with a smoothing parameter *α* is to replace the “+” function of (17) to obtain the following smooth support vector machine (SSVM) with a linear kernel:

(19) $$\begin{array}{c} \underset{w,b}{\min}\;\frac{1}{2}\left({||w||}^{2}+b^{2}\right)+\frac{C}{2}\overset{m}{\underset{i=1}{\sum }}p{\left(\left\{1-y_{i}\left(w^{t}x_{i}+b\right)\right\},\alpha \right)}^{2}. \end{array}$$

For specific data sets, an appropriate nonlinear mapping *x* ↦ *ϕ*(*x*) can be used to embed the original *R* ^*n*^ features into a Hilbert feature space *F*, *ϕ* : *R* ^*n*^ ↦ *F*, with a nonlinear kernel *K*(*x*_*i*_, *x*_*j*_) ≡ *ϕ*(*x*_*i*_)^*t*^ *ϕ*(*x*_*j*_). Thus, (19) can be extended to the SSVM with a nonlinear kernel:

(20) min⁡w,b  12(||w||2+b2)  +C2∑i=1mp({1−yi(∑j=1mvjK(xi,xj)+b)},α)2,

where ∑_*j*=1_^*m*^ *v*_*j*_ *K*(*x*_*i*_, *x*_*j*_) + *b* is the nonlinear SSVM classifier. The coefficient *v*_*j*_ is determined by solving an optimization problem (20) and the data points with corresponding nonzero coefficients.

With the principle of SSVM, we can formulate the interval linear regression model as follows:

(21) min⁡a¯,c¯,d¯ 12(a¯ta¯+c¯tc¯+d¯td¯+b2)+C2∑i=1mp({1−yi(wtxi+b)},α)2s.t. a¯xi+c¯|xi|≤yi+eia¯xi−c¯|xi|≥yi−eia¯xi+c¯|xi|+d¯|xi|≥yi+eia¯xi−c¯|xi|−d¯|xi|≤yi−eii=1,2,…,m,

where $\bar{a}$, $\bar{c}$, and $\bar{d}$ are the collections of all *a*_*i*_, *c*_*i*_, and *d*_*i*_, *i* = 1,2,…, *m*, respectively.

Given (21), the corresponding Lagrangian objective function is

(22) L≔12(a¯ta¯+c¯tc¯+d¯td¯+b2) +C2∑i=1mp({1−yi(wtxi+b)},α)2 −∑i=1mλ1i(yi+ei−a¯xi−c¯|xi|) −∑i=1mλ2i(a¯xi−c¯|xi|−yi+ei) −∑i=1mλ3i(a¯xi+c¯|xi|+d¯|xi|−yi−ei) −∑i=1mλ4i(yi−ei−a¯xi+c¯|xi|+d¯|xi|),

where *L* is Lagrangian and *λ*_1*i*_, *λ*_2*i*_, *λ*_3*i*_, and *λ*_4*i*_ are Lagrange multipliers. The idea to construct a Lagrange function from the objective function and the corresponding constraints is to introduce a dual set of variables. It can be shown that the Lagrangian function has a saddle point with respect to the primal and dual variables in the solution [29].

The Karush-Kuhn-Tucker (KKT) conditions that the partial derivatives of *L* with respect to the primal variables $\left(\bar{a},\bar{c},\bar{d}\right)$ for optimality

(23) ∂L∂a¯=0⟹a¯=−∑i=1m(λ1i−λ2i−λ3i+λ4i)xi,∂L∂c¯=0⟹c¯=−∑i=1m(λ1i+λ2i−λ3i−λ4i)|xi|,∂L∂d¯=0⟹d¯=∑i=1m(λ3i+λ4i)|xi|.

Substituting (23) in (22) yields the following optimization problem:

(24) max⁡ 12(∑i,j=1m(λ1i−λ2i−λ3i+λ4i)×(λ1j−λ2j−λ3j+λ4j)xitxj+∑i,j=1m(λ1i+λ2i−λ3i−λ4i)×(λ1j+λ2j−λ3j−λ4j)|xi|t|xj|−∑i,j=1m(λ3i+λ4i)(λ3j+λ4j)|xi|t|xj|+b2)+C2∑i=1mp({1−yi(wtxi+b)},α)2s.t. λ1i,λ2i,λ3i,λ4i≥0.

Similarly, we can obtain the interval nonlinear regression model by mapping *x* ↦ *ϕ*(*x*) to embed the original *R* ^*n*^ features into a Hilbert feature space *F*, *ϕ* : *R* ^*n*^ ↦ *F*, with a nonlinear kernel *K*(*x*_*i*_, *x*_*j*_) ≡ *ϕ*(*x*_*i*_)^*t*^ *ϕ*(*x*_*j*_) as discussed in Section 2.2. Then we obtain the optimization problem as (25) by replacing **x**_*i*_^*t*^ **x**_*j*_ and |**x**_*i*_|^*t*^ | **x**_*j*_| in (24) with *K*(**x**_*i*_, **x**_*j*_) and *K*(|**x**_*i*_ | , |**x**_*j*_|), respectively:

(25) max⁡ 12(∑i,j=1m(λ1i−λ2i−λ3i+λ4i)×(λ1j−λ2j−λ3j+λ4j)K(xi,xj)+∑i,j=1m(λ1i+λ2i−λ3i−λ4i)×(λ1j+λ2j−λ3j−λ4j)K(|xi|,|xj|)−∑i,j=1m(λ3i+λ4i)×(λ3j+λ4j)K(|xi|,|xj|)+b2)+C2∑i=1mp({1−yi(∑j=1mvjK(xi,xj)+b)},α)2s.t. λ1i,λ2i,λ3i,λ4i≥0.

## 4. Numerical Example

To illustrate the methods developed in Section 3, the following example is presented.

**Figure 2:**
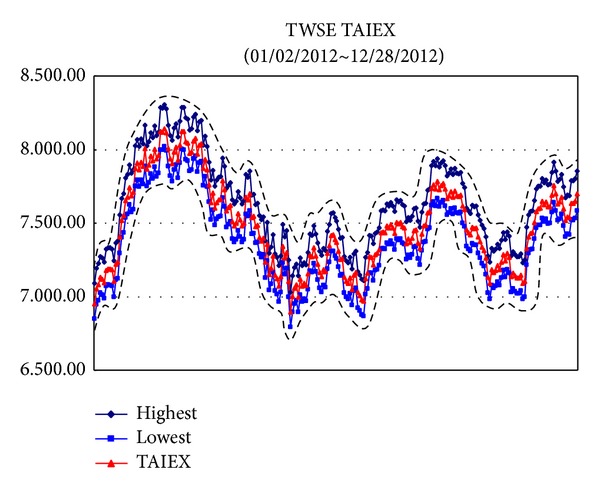
TAIEX [26] from 01/02/2012 to 12/28/2012.

**Figure 5:**
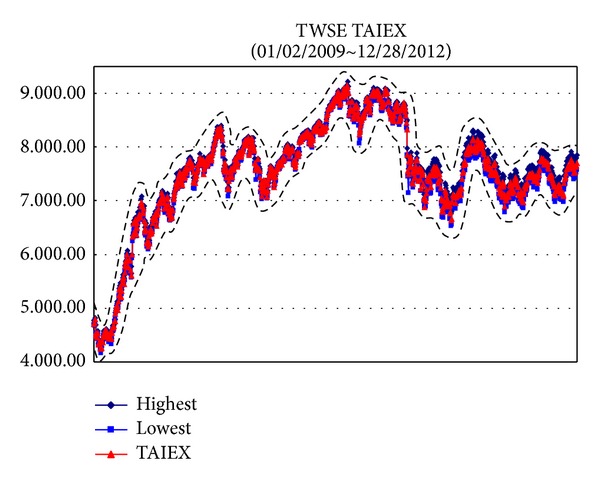
TAIEX [26] from 01/02/2009 to 12/28/2012.

*Example*. To illustrate the proposed methods dealing with big data sets, we use the data sets from Taiwan Stock Exchange Capitalization Weighted Stock Index (TAIEX) [26] which included the highest, lowest, and closed data and the ranges are from 01/02/2012 to 12/28/2012, from 01/02/2011 to 12/28/2012, from 01/02/2010 to 12/28/2012, and from 01/02/2009 to 12/28/2012, respectively. For these data sets, the Gaussian kernel [10] is used where *σ* = 2.5 and the regularization constant *C* = 300. The results are illustrated from Figure 2 to Figure 5.

The comparison is shown by using the measure of fitness [15] as (26), which defines how closely the possibility output for the *j*th input approximates the necessity output for the *j*th input. Consider

(26) $$\begin{array}{c} \varphi_{Y}\left(x_{i}\right)=\frac{1}{q}\overset{q}{\underset{j=1}{\sum }}\frac{c_{0}+\sum_{i=1}^{n}c_{i}\left|x_{ij}\right|}{c_{0}+\sum_{i=1}^{n}c_{i}\left|x_{ij}\right|+d_{0}+\sum_{i=1}^{n}d_{i}\left|x_{ij}\right|}, \end{array}$$

where *q* is a sample size and 0 ≤ *φ*_*Y*_ ≤ 1.

Table 1 presents the proposed methods with a Gaussian kernel along with the results computed by Tanaka and Lee [15], Hong and Hwang [16], and Huang [21]. We can find that the proposed methods are more efficient than other methods.

## 5. Conclusions

In this paper, we collaborate interval regression with SSVM to analyze big data. In addition, the soft margin method is proposed to modify the excursion of separation margin and to be effective in the gray zone. The main idea of SSVM is solved by a fast Newton-Armijo algorithm and has been extended to nonlinear separation surfaces by using a nonlinear kernel technology. The experiment results show that the proposed methods are more efficient than existing methods. In this study, we estimate the interval regression model with crisp inputs and interval output. In future works, both interval inputs-interval output and fuzzy inputs-fuzzy output will be considered.

## Figures and Tables

**Figure 3:**
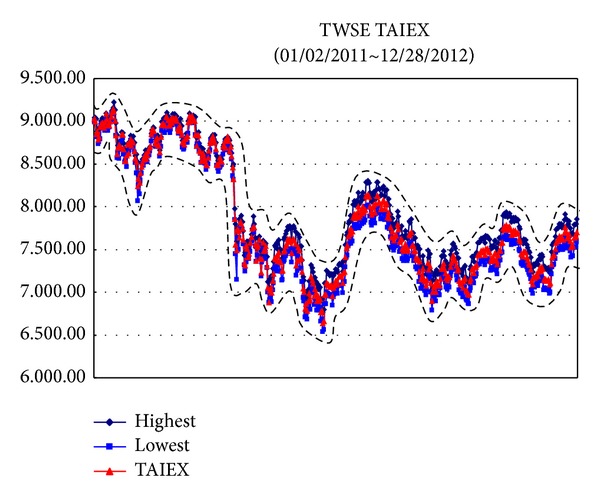
TAIEX [26] from 01/02/2011 to 12/28/2012.

**Figure 4:**
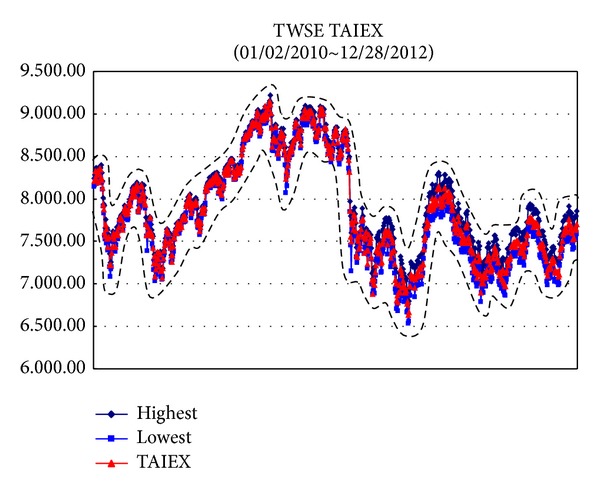
TAIEX [26] from 01/02/2010 to 12/28/2012.

**Table 1:** Comparison results of the measure of fitness.

	Tanaka and Lee [15]	Hong and Hwang [16]	Huang [21]	Proposed methods
*φ*_*Y*_ (Figure 2)	0.1404	0.1395	0.1412	0.1354
*φ*_*Y*_ (Figure 3)	0.1573	0.1562	0.1581	0.1429
*φ*_*Y*_ (Figure 4)	0.1694	0.1658	0.1706	0.1583
*φ*_*Y*_ (Figure 5)	0.1714	0.1695	0.1723	0.1609
